# The (dis-)connection between selection research in sports and business literature – a citation network analysis

**DOI:** 10.3389/fpsyg.2025.1604108

**Published:** 2025-06-23

**Authors:** Birte Brinkmöller, Dennis Dreiskämper, Oliver Höner, Bernd Strauss

**Affiliations:** ^1^Department of Sport and Exercise Psychology, Institute of Sport and Exercise Sciences, University of Münster, Münster, Germany; ^2^Department of Sports Psychology, Institute for Sport and Sport Science, Dortmund University, Dortmund, Germany; ^3^Department of Sport Psychology and Research Methods, Institute of Sports Science, Eberhard Karls University of Tübingen, Tübingen, Germany

**Keywords:** bibliometric study, CNA, interdisciplinarity, personnel selection, talent identification

## Abstract

Sports associations and companies share a common goal of identifying and selecting top talent, prompting a growing interest in refining their selection processes. Recent decades have witnessed increased attention to predictive valid constructs (e.g., motivation) and predictive assessment methods (e.g., questionnaires). Leveraging an extensive history and empirical evidence from personnel selection research in business, a cross-contextual knowledge transfer could offer potential benefits for both sports and business. To examine the utilization of information between sports and the business context, this study explores the connection between these contexts. Analyzing 20,492 articles from SCOPUS and Web of Science databases, we followed PRISMA guidelines and conducted a citation network analysis (*n* = 940 articles). Results reveal limited interconnectivity (3,728 links), with only six articles cited between contexts: three sports articles cited business articles and vice versa. Sports articles referred to business research for talent definition, skill assessment, and methodological concerns. Contrarily, the business domain primarily used sports research as examples and for individual aspects of talent development models. This study highlights the potential for points of connection, including requirement analysis, skill assessment, and research on predictive validity. Future research should systematically explore shared and underrepresented topics in both contexts, such as the use of, for example, situational judgement tests in sports to enhance talent selection in sports, drawing insights from the business domain.

**Systematic review registration:**

https://aspredicted.org/366x-7cfd.pdf.

## 1 Introduction

The systematic process of identifying and selecting high-potential individuals is a critical factor in optimizing organizational performance across various professional contexts, including sports associations and corporate environments. The quality of selection processes and decisions may be decisive for the competitiveness and innovation of both, sporting teams and companies. Through talent selection decisions, sport associations and/or clubs try to find the most talented athletes to not only provide them with training programs but also to gain a competitive advantage against other clubs (e.g., soccer youth academies) or other nations (selection for an international squad). In companies, the same picture emerges so that the goal is to not only enhance job performance, but also organizational performance and competitive advantage (Ployhart et al., 2017). Thereby, the selection decision is embedded in a larger process, which in sports also involves the identification of athletes and in companies the recruitment of potential employees (Williams and Reilly, 2000; Lievens et al., 2021). At first glance, there are similarities between the two contexts, including the goal of selecting suitable individuals and the use of valid and reliable methods for selection. However, there are also differences, such as the age of the individuals when selection takes place. Recent research by Parra-Martinez and Wai (2023) provides further support for the relevance of comparing talent identification processes across fields, as their analysis demonstrates shared theoretical and methodological approaches in diverse contexts, including, for example, sports and business. Their findings underscore the potential of interdisciplinary insights to advance understanding in individual fields.

Additional support for the value of comparative studies comes from research in other contexts, such as the integration of psychology and marketing (Donthu et al., 2021). Moreover, studies comparing sport and business have begun to explore overlapping constructs, such as trust in performance-oriented teams, further emphasizing the applicability of such comparisons (McGuire and Martin, 2023).

Based on the similarities, it can be reasonably assumed that the methodological and theoretical-conceptual approaches to talent selection in the two contexts may have a scholarly connection. Such a linkage could facilitate the application of findings that have already been researched in one field but have not yet been transferred to the other. To date, however, to the author's knowledge, no further studies exist that have systematically investigated the interrelationship between the two fields of research through an overarching, systematic literature review, with a particular focus on the selection process of athletes and employees.

### 1.1 Talent selection in sports

The selection process of athletes in sports is mainly characterized by its implementation in larger programs. The overarching aim of talent identification is to identify and select young athletes for promotion programs who have the greatest potential to succeed in elite sports (Vaeyens et al., 2009). According to Williams and Reilly (2000), early talent development includes talent detection, talent identification, talent selection and talent development. Talent detection refers to the process of discovering individuals with potential who are not currently engaged in the specific sport; talent identification refers to the process of recognizing those athletes who are already involved in the sport and have the potential to become successful. This stage is part of talent development, implying that athletes are placed in a suitable learning environment to develop their potential. Talent selection is an ongoing process that focuses on identifying athletes at different stages who exhibit the required performance levels for inclusion in a specific team or squad. This process entails selecting the most suitable individual or group (Williams and Reilly, 2000).

The ongoing decisions of selection and deselection are one of the biggest challenges for coaches (Capstick and Trudel, 2010). They are asked to evaluate the potential of young athletes and predict their future performance, which is highly challenging (Bergkamp et al., 2019). Decisions are made based on either an objective approach, a subjective approach or both (Bar-Eli et al., 2024). The latter has been perceived as the most promising approach to predict an athlete's future success, given the large amount of information, which is therefore most relevant for decision-makers. While the objective approach typically includes test scores, which assess motor skills or psychological aspects (Bar-Eli et al., 2024; Lath et al., 2021), the subjective approach refers to the *coach's eye* (Lath et al., 2021). Coaches make decisions on selection or de-selection based on their overall impression and observations (Roberts et al., 2019). Therefore, the information regarding clear criteria or the weighting of different aspects remains unclear (Bergkamp et al., 2022a).

By aiming to create a comprehensive evaluation of an athlete's potential for future success, various talent predictors, based on talent development models (e.g., Gagné, 2010; Ullén et al., 2016) are assessed alongside subjective coach impressions. Selection criteria include anthropometric characteristics as well as physical and physiological attributes, which are indisputable for performance (Lidor et al., 2009) and simple to assess. Cognitive and psychological factors, as well as technical and tactical skills (Johnston et al., 2018; Faber et al., 2016), especially sport-specific technical skills, have also been found to be predictive for future success in a variety of sports (i.e., Elferink-Gemser et al., 2007; Huijgen et al., 2014; Höner et al., 2017) and thus offer a potential for talent selection (Koopmann et al., 2020). Furthermore, sport-specific perceptual-cognitive performance factors such as decision-making seem to be critical for future performance in some sports (e.g., soccer; Kannekens et al., 2011). Within the most researched sport, soccer, the motivational component “hope for success” (Zuber et al., 2015) as well as task orientation are positively associated with future performance and negatively associated with “fear of failure” (Höner and Feichtinger, 2016). Furthermore, competition orientation (Höner and Feichtinger, 2016) goal orientation (Zuber et al., 2015; Höner and Feichtinger, 2016) and goal commitment (Van Yperen, 2009) show relationships with future soccer success. Regarding non-sport-specific psychological characteristics, goal management skills show moderate evidence to predict future performance in racquet sports (Faber et al., 2016). Höner and Feichtinger (2016) also found the volitional competency self-optimization, self-efficacy, specific and general physical self-concept and worry, as a competition anxiety component, to be related to future success in soccer. Thus, the existing literature indicates that talent selection is an extensive and dynamic field that varies in scope and content across sports and countries, relies on diverse methods and determinants, and continues to evolve.

### 1.2 Talent selection in business

Similar to talent selection in sports, the aim of personnel selection in the business context is 2fold: enhancing organizational performance and competitive advantage on a macro level, while improving individual job performance on a micro level (Ployhart et al., 2017). Individual job performance is traditionally conceptualized as comprising task performance—the execution of job-specific responsibilities—and non-task performance, such as organizational citizenship behavior (OCB) and counterproductive work behavior (CWB; Borman and Motowidlo, 1997; Dalal, 2005; Lievens et al., 2008; Organ and Ryan, 1995; Rotundo and Sackett, 2002).

Building on this understanding of performance outcomes, the hiring process typically involves a series of interconnected steps: identifying and attracting potential candidates during recruitment, systematically evaluating their suitability through selection methods, and ultimately integrating them into the organization. According to the American Psychological Association (APA), selection refers to the process of selecting employees best suited for particular jobs by using procedures such as assembling and analyzing biographical data, conducting employment interviews, and administering employment tests.

To assess the effectiveness of these methods, meta-analytic findings have consistently shown that job-specific methods are among the most effective predictors of job performance (Sackett et al., 2022). Thereby, structured interviews exhibit the highest validity estimate in terms of effect sizes (ρ = 0.42), followed by job knowledge tests (ρ = 0.40), empirically keyed biodata (ρ = 0.38), and work sample tests (ρ = 0.33; Sackett et al., 2022). All of these are job-specific measures. General cognitive ability tests (ρ = 0.31 in Sackett et al. (2022); ρ = 0.22 in Berry et al. (2024), integrity tests (ρ = 0.31) and personality-based emotional intelligence (ρ = 0.30) also rank among the strongest predictors, considered psychological constructs. Assessment centers (AC; ρ = 0.29) and situational judgment tests (SJT; ρ = 0.26) are still in the Top 8. These findings suggest that a strong predictive relationship may be supported by a closer match between the predictor and the criterion (Sackett et al., 2022).

However, it is important to recognize that while some methods measure a specific construct, e.g., cognitive ability tests measuring cognitive ability, different methods inherently target distinct constructs. Interviews, for instance, often evaluate candidates based on job-related content, their performance during the interaction, and even demographic or personal characteristics (Huffcutt et al., 2001). Interview questions can encompass general traits (e.g., cognitive ability, personality, values), experiential factors (e.g., education, prior experience, training), and job-specific elements (e.g., knowledge, skills, motivation; Huffcutt et al., 2001). The reliability and validity of interview outcomes are thereby heavily dependent on conducting a thorough job analysis to ground questions in job-relevant criteria.

In terms of psychological predictors, general mental ability (GMA) has long been recognized as a robust predictor of task performance (Berry et al., 2024). However, its relationship with non-task performance behaviors, such as CWB or OCB, is less pronounced (Salgado et al., 2003). GMA shows nearly zero correlation with CWB and only modest positive correlation with OCB. In contrast, personality traits—especially those defined by the five-factor model—exhibit stronger relevance for non-task behaviors (Gonzalez-Mulé et al., 2014). The differentiation between OCB and CWM as performance outcomes underscores the importance of understanding performance as multifaceted, with task and non-task performance being influenced by distinct predictors. Consequently, recent research has shifted toward incorporating personality assessments (Ryan and Ployhart, 2014), candidate interests (Van Iddekinge et al., 2011), and constructs like emotional intelligence (Christiansen et al., 2010) and integrity. While the latter's utility may vary depending on theoretical assumptions, these measures provide a nuanced approach to predicting both job performance and potential turnover. They are, therefore, key representative characteristics in the selection of companies. In summary, research on personnel selection is well-established and provides strong evidence for the effectiveness and predictive validity of methods and constructs.

### 1.3 Comparison of sports and business selection processes

As outlined above, selection procedures and methods across the contexts of sports and business reveal prominent parallels, as well as distinctive characteristics, which justify scientific integration. A greater empirical evidence base seems to be evident in the business sector, which could lead to possible added value for the sports sector through a comparison of the two contexts.

Key differences emerge in certain aspects of the selection process between the two contexts. While the selection of athletes in sports typically begins at an early age (approximately under 12 years; Ford et al., 2020), accounting for maturational aspects and developmental potential (Vaeyens et al., 2008), selection in companies typically takes place at discrete career intervals, beginning after school with an emphasis on current performance indicators (Dries, 2013). Furthermore, in sports, physical and physiological aspects play a dominant role (Baker et al., 2020) while requirements for job performance in companies are largely based on cognitive aspects (Berry et al., 2024).

Despite these differences, there are also striking commonalities, which may indicate a possible linkage between both contexts exists: from a general developmental perspective, selection research in both contexts stems from the fundamental discipline of psychology (Parra-Martinez and Wai, 2023). Meta-analytical and systematic review findings show remarkable convergences regarding selection parameters between sports (e.g., Johnston et al., 2018) and organizational selection processes (e.g., Silzer and Church, 2009). Both contexts emphasize a thorough analysis of the requirements, followed by a multi-dimensional assessment, including domain-specific performance characteristics, e.g., cognitive and physiological aspects, as well as psychological attributes as predictors of future performance. The importance of multidimensionality for successful performance has been shown in both contexts [e.g., Macnamara et al. (2010) in sports; Cortina and Luchman (2013) in business]. Additionally, a combination of objective and subjective measurements is used to Bar-Eli et al. (2024) and Highhouse (2008). Regarding this decision, both contexts face similar methodological challenges, including the validity of predictions, the reliability of methods and constructs, and the integration of subjective and objective selection criteria.

Consequently, the context-specific nature of selection criteria may render discussions at this level somewhat redundant. However, a systematic analysis of methodological approaches across different contexts, along with the exploration of promising procedures and the assessment of non-contextual constructs (e.g., psychological factors), can offer valuable insights into the evolution and refinement of selection processes within both domains. This analysis centers on evaluating the degree of convergent development in systematic selection processes across these ostensibly distinct yet methodologically interconnected contexts. Nevertheless, it remains unclear whether the employed methods and empirically validated relationships between constructs and performance are solely derived from and confined to their original contexts. Furthermore, it is uncertain whether identical methods and constructs are utilized across both contexts and subjected to cross-contextual adaptation. Currently, evidence suggests minimal overlap between the two contexts at both the scientific and methodological levels.

### 1.4 Aim of the study

In sports, as in business, represent complex systems of selection, and fundamentally aim to identify, select and develop people. Despite some distinct aspects in their selection processes, such as the predominant focus on cognitive aspects in business and physiological aspects in sports, some of the same methods are used and the same constructs are recorded. Furthermore, both contexts call for a strict differentiation between methods and constructs (Bergkamp et al., 2019; Arthur and Villado, 2008). Based on the similarities, we assume that a scholarly linkage between the two contexts may be beneficial for both gaining knowledge about empirically based selection aspects and transferring knowledge. Especially the large volume of empirical findings in the business context can make a particular contribution to sport by possibly integrating methods or assessing valid constructs. So far, the extent of their potential interconnectedness remains an open question. Our research, therefore, seeks to systematically explore whether meaningful knowledge transfer or intellectual convergence exists between these contexts.

Previous research has not systematically examined the potential relationship between sports and business research, particularly in the context of talent selection. Specifically, a comprehensive systematic review of the articles included in prior analyses has not been conducted (Parra-Martinez and Wai, 2023). If an association exists between the two contexts, we assume a predominantly knowledge transfer from business to sports contexts, on the one hand, and that the fundamental discipline of psychology serves as a link between the two contexts through general theories, models, and psychological aspects, on the other hand.

We aim to provide initial insights into the possible link between the two contexts, with the intention of understanding whether parallel strands exist between the research lines. The aim of the study is therefore to assess the extent to which the talent selection literature in business is connected to the talent selection literature in sports, based on the approach of a configurative review. We aim to provide a thorough picture of the fields by including aspects of aggregate reviews, such as a priori inclusion criteria (Gough et al., 2012). We aim to 1a) assess the possible link between the two contexts and (1b) evaluate whether they potentially share a common theoretical foundation through the foundational discipline of psychology. We further aim to (2) identify articles that cross the border of fields by also influencing the other field (i.e., sports publications for business and vice versa). To assess the scholarly linkage, we employed a citation network analysis, complemented by a preceding systematic literature review to enhance our understanding of previous scientific work (Parra-Martinez and Wai, 2023). We also aim to assess the content structure of both fields by extracting (3) the most influential articles and (4) the most influential authors in each field.

## 2 Methods

To answer the research questions, we conducted a citation network analysis (CNA), following the guidelines by McLaren and Bruner (2022) regarding the research question of the interconnectedness, timing, systematic search, descriptive statistics, basic network metrics and open science frameworks. Compared to other quantitative research designs, such as meta-analysis and systematic reviews, CNA “seeks to map the scientific structure of a field of research as a function of citation practices” (McLaren and Bruner, 2022). With this, citation structures are made visible between articles to see which findings are used as a foundation.

### 2.1 Data extraction and preparation

As a first step of the CNA a systematic literature search was conducted using the PRISMA guidelines (Page et al., 2021). Articles were searched in the SCOPUS database from Elsevier and the Web of Science Core Collection (cf.) (Parra-Martinez and Wai, 2023; Bruner et al., 2009). With both, we covered the two biggest databases for peer-reviewed literature and the most powerful research engine for citation data. Search criteria were limited to (1) articles and reviews, (2) published in the source type journal, and (3) English language literature, regardless of publication date. Owing to the substantial volume of articles and the emphasis placed on quality, the decision was made to exclude conference papers (by possibly presenting preliminary results) and abstracts (without a complete reference list) from the study. Further, accessibility to a wider audience as well as the potential for duplicate inclusion of findings, monographs and dissertations/theses were excluded. The search was limited to the subject areas “business, management, and accounting (all),” “economics, econometrics, and finance,” “psychology (all)” and “undefined,” given the vast number of possible articles. To provide a holistic representation of the connection between domains, the limitations ensure that the found articles have a direct relevance to the topics pertinent to the research question. Additionally, including “undefined” allows for a broader search to capture potentially relevant articles that may not fit clearly into any of the other categories. As “sports” was not a subject area itself, articles within this context fell under “undefined” or “psychology (all).”

The following terms and operators were used for the search based on their presence in the abstract/title/keyword (OR within search strings, AND between search strings): assessment AND center OR talent^*^ OR “personnel selection” OR recruit^*^ OR “talent development OR “talent identification” OR giftedness OR “talent selection” OR “talent management” OR screening) AND ((organization OR industry OR “high performance” OR business) OR (sport^*^ OR “elite sport^*^”)).

In contrast to the clean merged dataset of Parra-Martinez and Wai (2023; *n* = 2,502 articles), our dataset encompassed 18,835 articles as of June 30th, 2022. This significant difference in the number of articles is the result of a broader literature search. By including various search terms specific to both domains (e.g., *talent selection* for sports, or *personnel selection* for business), our search was more comprehensive without limiting our search terms to articles including develop^*^.

Titles were screened in a double-blind fashion by five independent raters using Rayyan (Ouzzani et al., 2016), manually without AI. All raters have a background in sports sciences; the first rater also has a background in I/O psychology. Before screening, all raters were given a detailed introduction to both contexts. Additionally, the first rater was always available to answer any queries. The first author rated all titles, and four raters were randomly assigned to each title, resulting in two ratings per title. To achieve the study's aim, studies were included in the network analysis if they addressed the overall topic of talent selection, talent identification, or talent management in the context of sport or business. Furthermore, studies had to contain at least one of the following:

*Methods*: studies must have described, evaluated, or compared any method used for talent or personnel selection in the context of sport or business.

*Procedure/process:* studies must have described, evaluated, or compared the selection process (in sport or business) within the field or with any other field.

*Constructs*: studies must have described or evaluated constructs using any performance measurement that may be used as a performance predictor. The population consisted of athletes or employees, regardless of their performance level.

*Reviews:* reviews must have addressed the topic of talent selection, talent identification, or talent management (e.g., definitions of talent and talent management), which may impact the process in some way.

**Figure 1:**
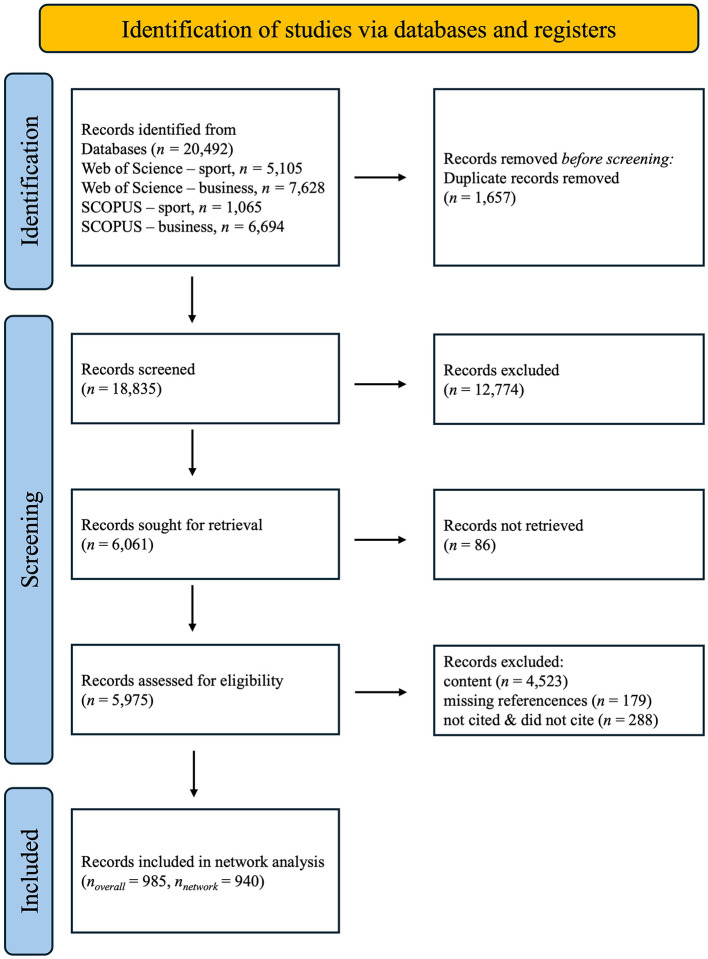
PRISMA Flow chart (Preferred reporting items for systematic reviews and meta-analysis; Page et al., 2021). This figure shows the number of records that were collected and the number of eligible records after the screening process.

The rationale for including all talent selection research, regardless of the level of sports, position, and age of applicants, is based on the fact that although the demands and requirements of different levels and ages may vary, there is an overlap in the underlying factors contributing to talent selection. Furthermore, the inclusion of all levels and ages enhances the generalizability of the findings. Including all groups can enhance the transferability of knowledge across domains, with implications for one group that can be applied to another. This procedure (McLaren and Bruner, 2022) aligns with the guidelines by McLaren and Bruner (Arthur and Villado, 2008), who recommend being “more rather than less inclusive in terms of eligibility” (p. 10). After rating, articles which were included by at least one rater remained in the screening process [following McLaren and Bruner (2022) by being more inclusive; κ = 0.43]. For the abstract screening process the inclusion and exclusion criteria were narrowed to include only those articles that focus exclusively on talent selection, regardless of their focus on methods, theories, or criteria. Exclusion criteria focused on talent development and only descriptive differences between elite/non-elite. Further, articles focusing on the applicant's perception of the selection process were excluded. This approach aimed to provide a robust and accurate depiction of the connections within the domain of talent identification. Subsequently, the abstract screening, also in a double-blind fashion, was performed by the same independent raters (one rater rating all abstracts, four raters randomly distributed) for those articles which had abstracts available. The interrater reliability showed an acceptable level of agreement (κ = 0.54). To address any discrepancies, all conflicts were thoroughly discussed and resolved collaboratively among pairs of raters. Only articles which seemed eligible for both raters after discussion remained. The screening yielded *n* = 1,452 eligible articles (nodes) for which references were downloaded (Figure 1). Links, which represent the connections (references) between articles, were developed manually using Microsoft Excel. An ID was assigned to each article, and the citation of each article was searched in the network and replaced by the corresponding ID. Only those articles which were cited or cited by another article within the scope were included in the citation network analysis.

### 2.2 Citation network procedure and analysis

To conduct the citation network analysis, *n* = 985 included articles were classified into the three categories “business,” “sport,” and “general psychology.” In the first round, they were assigned based on the journal in which they were published. Both the journal article and the journal category classification of SCImago JR were used. SCImago JR is a freely accessible journal ranking portal and is modeled on the SCOPUS database. When dividing the articles, we started with the field of “sport,” then “business,” and lastly “general psychology.” The category “general psychology” included articles which were not directly related to sports or business but dealt with the topic of selection in a psychological and general manner, such as predictive validity or specific constructs (e.g., association between motivation and performance). An exception to the allocation, however, were those articles that could be explicitly assigned to one of the fields based on their content orientation, although they were published in a journal of the other field (e.g., articles that were focused on the sports context but published in a business journal, based on the previous classification). Those articles were assigned to the category based on their content orientation, rather than the journal's orientation.

Visualizing the articles as a citation network was conducted using the package iGraph in R Studio (Version 2022.02.2). After removing the outliers (which had no connection to the overall network), 940 nodes and 3,728 links remained in the final CNA. Additionally, to assess the intellectual structure, a co-citation network analysis was conducted using *bibliometrix* in *biblioshiny* (Aria and Cuccurullo, 2017). A co-citation is present when two articles are both cited by another article. The analysis was conducted using a Louvain clustering algorithm (McGuire and Martin, 2023). As it was not possible to merge data from multiple databases into *bibliometrix*, as initially searched, we searched for all articles included in the citation network in Clarivate Analytics Web of Science (WoS) again. *N* = 893 (95% of the initial article population) articles were found (list of missing articles: Supplementary Table S4).

Next to visualizing the interconnectivity, we also measured the quantitative knowledge flow and the salient articles of each domain. For this purpose, the directional paths were divided into directions, i.e., “sport” citing “business,” “business” citing “general psychology,” etc. (resulting in six directions) and counted. To focus on the most salient articles, we measured the number of citations each article received within its category and eliminated those that were not only within the category but also between categories (within categories, the top 10 articles). With this analysis, we were able to determine whether there are prominent articles within one field that are also prominent within another field. To extract the most influential articles, centrality measures (eigenvector, betweenness, degree, in-degree) were calculated using iGraph from R Studio (Version 2022.02.2). Degree-centrality scores show the number of links held by each node, i.e., the number of times an article is cited by, or cites, another article in the scope (Hancock et al., 2015). The in-degree centrality scores represent the number of inbound links and measure the prominence of articles in terms of their citation by other articles (Moore et al., 2005). To expose the influence that an article has in the network, the eigenvector centrality was calculated. Eigenvector centrality depends on the number of direct and indirect connections as well as on their connection to other articles (Bonacich, 1987). In addition, betweenness centrality, representing the number of cases in which a node can be reached by the shortest path between two other nodes (Borgatti et al., 2018) was measured. Articles with a high betweenness centrality act as bridges between nodes in a network, enabling the flow of information.

In line with Parra-Martinez and Wai (2023) top-ranked authors of the finally retrieved studies were calculated by the number of articles published within the network as an indicator of productivity. The fractionalized publication score was calculated by summing all proportioned credits assigned to an author (i.e., for a publication with five authors, each author receives 0.2 points).

## 3 Results

A summary of the articles' characteristics is shown in Table 1. Articles were published in 226 different journals (*M* = 4.2; *SD* = 8.2; *min* = 1; *max* = 66, *max*_*s*_ = *Journal of Sport Sciences, n* = 66; *max*_*b*_ = *International Journal of Selection and Assessment, n* = 58). The most cited sports articles were published in the year 2015 (*n*_*citations*_ = 177), while the most cited business articles were published in 2003 (*n* = 181).

**Table 1:** General information of articles included in network.

**Description**	**Results**
**Core information about data**	
Timespan	1977–2022
Sources (journals)	226
Documents	940
Annual growth rate	17.60%
Document average age	10.9 years
Average citations per document	3.97
References	3,728
**Authors**	
Total authors	2,146
Unique authors of single-authored documents	78
Total first authors	729
**Author collaboration**	
Single-authored documents	88
Co-authors per document	2.27
**Field**	
Business (# of articles)	539
Sports (# of articles)	362
Psychology (# of articles)	39
Business (# of citations)	1,896
Sports (# of citations)	1,745
Psychology (# of citations)	87

Within the included articles, more than half of all articles (*n* = 660) have been published since 2010 with an increased number per decade (*n*_1970 − 1979_ = 1, *n*_1980 − 1989_ = 10, *n*_1990 − 1999_ = 67, *n*_2000 − 2009_ = 202, *n*_2010 − 2019_ = 500, *n*_*since*2020_= 160). The highest number of publications within business was in 2021 (*n* = 52), while in sports it was in 2017 and 2018 (*n* = 39).

### 3.1 Scholarly linkage between sports and business literature

**Figure 2:**
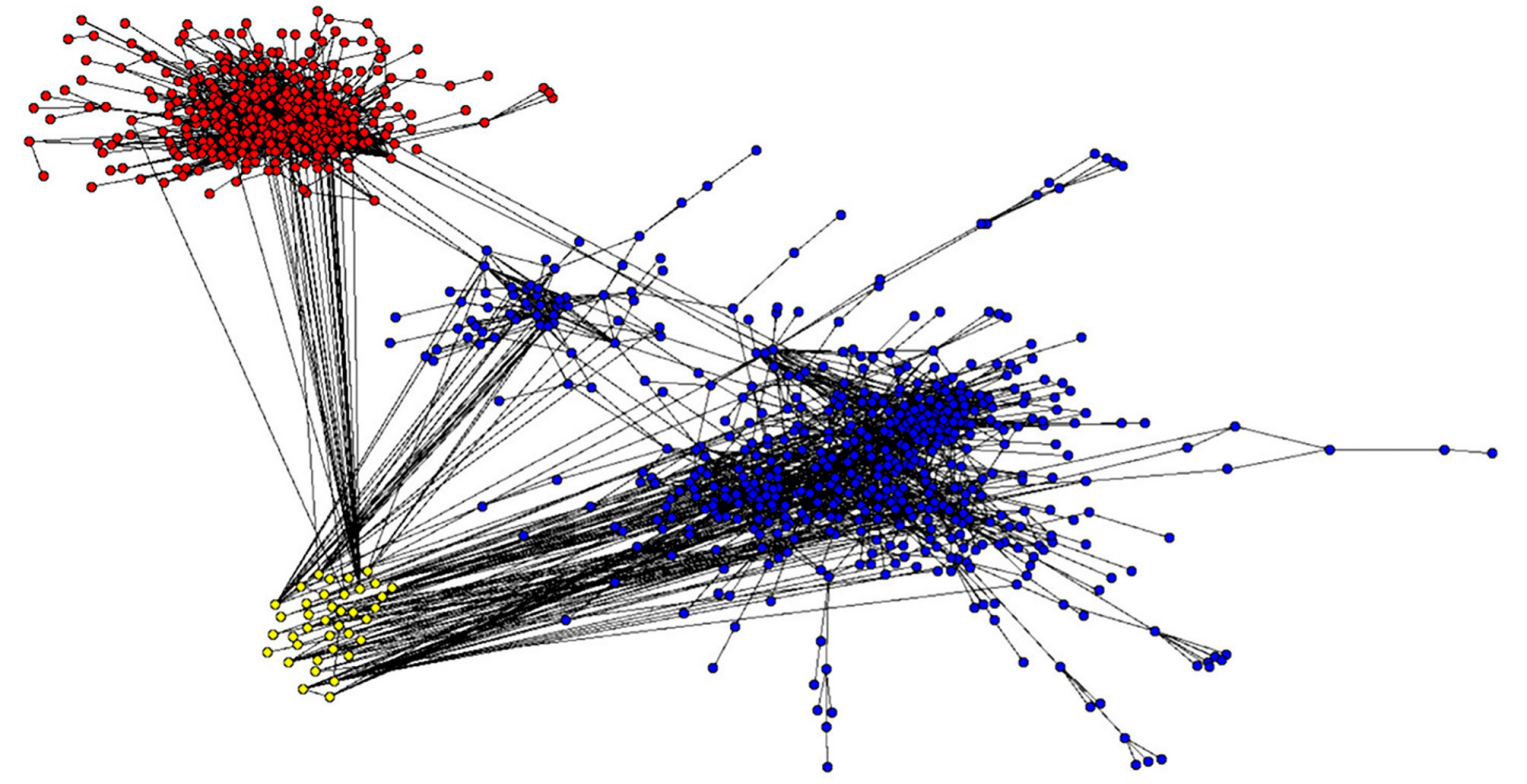
Visualization of talent selection literature citation network.

To address Research Question (RQ) 1a and RQ1b, i.e., the extent to which the sport and business literature, as well as general psychology literature, is connected, as well as RQ 2, identifying possible links between both contexts, we calculated the distribution of cited articles by the different fields (Table 2) and visually mapped the citation network (Figure 2). The citation network displays the interconnectivity of these communities, with each article represented by a node and every citation represented by a link. Thus, networks with a higher number of links indicate high interconnectivity, whereas fewer links indicate a lower level of interconnectivity or even a lack of interconnectivity. As shown in Figure 2, the sports articles (red) and the business articles (blue) are well-connected within their respective fields; however, they mostly remain separate from each other, except for some isolated links. The links are shown in Table 3. Four out of six articles which cite the other context were published in or after 2018, while one article was published in 2014. The earliest published article citing the other context (business citing sport) was published in the year 2000. Within the business scope, articles are further divided into two clusters, referred to as the main cluster (on the right-hand side) and the subcluster (on the left-hand side). The yellow nodes, which indicate articles belonging to the “general psychology” category, seem to be interspersed among the other domains, mainly within the business literature. This visualization shows the lack of communication between these two fields without general psychology acting as a bridge. The subcluster of the business field includes *n* = 53 articles. Within this cluster, the most prominent articles are Al Ariss et al. (2014), with *n* = 22 citations, (Nijs et al., 2014; *n*_*cited*_ = 15) and Thunnissen (2016; *n*_*cited*_ =10). *N* = 41 articles deal with talent management (i.e., the strategic identification, development, and retention of individuals to enhance organizational performance), defined by either including “talent management” in the title or as a keyword. The remaining articles address the role of technology (Nijs et al., 2014; Thunnissen, 2016), equality (Schmidt and Hunter, 1998; Barrick and Mount, 1991), the role of and perception from managers (Hunter and Hunter, 1984; Judge and Ilies, 2002; Tett et al., 1991), leadership (Arthur et al., 2003), HR in the public sector (Barrick and Mount, 1991; Gaugler et al., 1987) and the definition and operationalization of talent (Nijs et al., 2014; Thunnissen, 2016).

**Table 2:** Distribution of the absolute and relative number of citations based on category of referencing articles and referenced articles.

**Cited by Paper category**	**Business (*n =* 1,896)**	**Sport (*n =* 1,745)**	**General psychology (*n =* 87)**
Business	*n* = 1,824; 96.2 %	*n =* 3; 0.17%	*n =* 59; 67.82%
Sport	*n =* 3; 0.16%	*n =* 1,729; 99.08%	*n =* 20; 22.99%
General psychology	*n =* 69; 3.64%	*n =* 13; 0.74%	*n =* 8; 9.2%

**Table 3:** Cross-referenced paper from sports and business.

**Business paper cited in sports literate**	**Cited by**
Nijs S, Gallardo-Gallardo E, Dries N, Sels L. A multidisciplinary review into the definition, operationalization, and measurement of talent. Journal of World Business. 2014 Apr;49(2):180–91.	Johnston K, Wattie N, Schorer J, Baker J. Talent identification in sport: A systematic review. Sports Medicine. 2018;48(1):97–109.
Robertson IT, Smith M. Personnel selection. J Occup Organ Psychol. 2001;74(4):441–72.	Bergkamp TLG, Niessen ASM, den Hartigh RJR, Frencken WGP, Meijer RR. Methodological issues in soccer talent identification research. Sports Medicine. 2019 Sep 1;49(9):1317–35.
Lievens F, Patterson F. The validity and incremental validity of knowledge tests, low-fidelity simulations, and high-fidelity simulations for predicting job performance in advanced-level high-stakes selection. Journal of Applied Psychology. 2011 Sep;96(5):927–40.	Den Hartigh RJR, Niessen ASM, Frencken WGP, Meijer RR. Selection procedures in sports: Improving predictions of athletes' future performance. Eur J Sport Sci. 2018;18(9):1191–8.
**Sport paper cited in business literature**	**Cited by**
Blakley BR, Quinones MA, Crawford MS, Jago IA. The validity of isometric strength tests. Pers Psychol. 1994;47(2):247–74.	Terpstra DE, Kethley RB, Foley RT, Limpaphayom WT. The nature of litigation surrounding five screening devices. Public Pers Manage. 2000 Mar 1;29(1):43–54.
Bailey R, Morley D. Toward a model of talent development in physical education. Sport Educ Soc. 2006 Aug;11(3):211–30.	Nijs S, Gallardo-Gallardo E, Dries N, Sels L. A multidisciplinary review into the definition, operationalization, and measurement of talent. Journal of World Business. 2014 Apr;49(2):180–91.
Vaeyens R, Güllich A, Warr CR, Philippaerts R. Talent identification and promotion programmes of olympic athletes. J Sports Sci. 2009 Nov;27(13):1367–80.	Finkelstein LM, Costanza DP, Goodwin GF. Do your high potentials have potential? The impact of individual differences and designation on leader success. Pers Psychol. 2018 Mar 1;71(1):3–22.

### 3.2 Co-citation network

**Figure 3:**
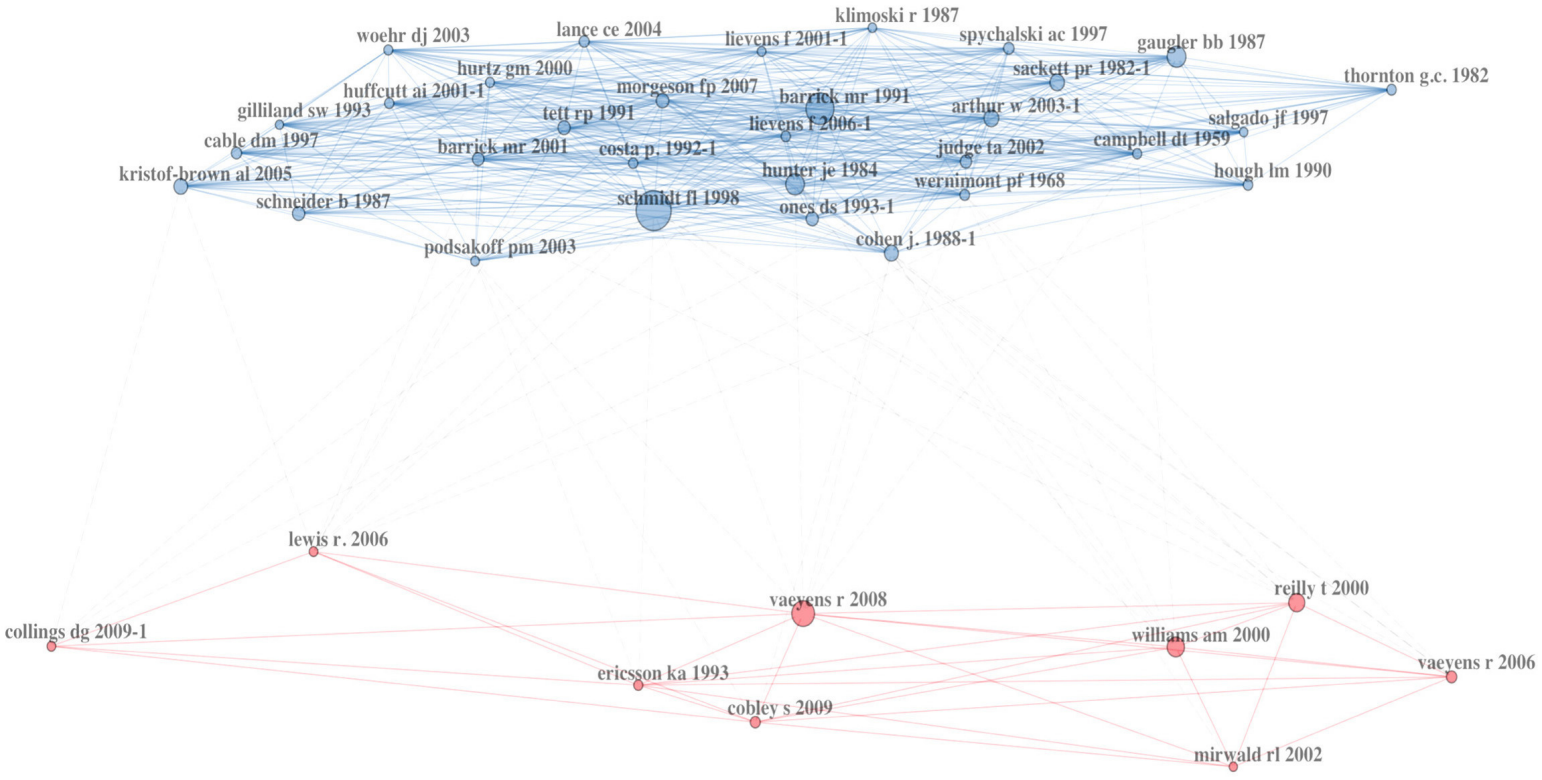
Co-citation network analysis.

The additional co-citation network analysis (Figure 3) supports the findings. The network is divided into two main clusters. The first cluster on the top (blue) is centered around Schmidt and Hunter (1998), Barrick and Mount (1991), and Hunter and Hunter (1984), focusing on the validity and reliability of various selection methods and their comparison. Additionally, articles dealing with personality traits (e.g., Judge and Ilies, 2002; Tett et al., 1991) as well as focusing on AC (Arthur et al., 2003; Gaugler et al., 1987) are located in this cluster. The co-citation of Cohen (1988) shows a slight connection to the second cluster due to its proximity to the cluster, as indicated by statistical information. Within the other cluster (red), sports articles are included, primarily related to Vaeyens et al. (2008), providing an overview of TID in sports and Reilly et al. (2000), a review of anthropometric and physiological predispositions for soccer. Further analyses of the network, e.g., author collaborations and co-occurrence of words, which exceed the scope of the article, can be found in the Open Science Framework (https://uni.ms/xbu2i).

### 3.3 Influential articles and authors

To answer RQ3 and RQ4, the most cited articles within the business literature and the sport literature are presented in Table 4 (Top 10 Articles: Supplementary Table S1). For the most influential authors (Table 5), degree centrality scores ranged from a minimum of 1 to a maximum of 143, with Vaeyens et al. (2008) having the highest score within the final scope. The mean degree centrality was *M* = 7.9 (*SD* = 9.7). The in-degree centrality ranged from min = 0 to max = 130 (Salgado et al., 2003) with a mean of *M* = 3.97 (*SD* = 8.18). The highest eigenvector centrality was also calculated for Vaeyens et al. (2008). The highest betweenness was measured for Robertson and Smith (2001). Centrality measures for the top 5 articles of each centrality measurement can be found in the Supplementary Table S2. Within the top 40 authors (Supplementary Table S3), *n* = 24 authors mainly published in the domain of sports, whereas *n* = 16 authors are allocated to the business domain.

**Table 4:** Top 3 cited paper including number of citations within sport and business literature (indegree centrality).

**Top 3 cited sports article**	**# citations**
Vaeyens R, Lenoir M, Williams AM, Philippaerts RM. Talent identification and development programmes in sport. Sports Medicine. 2008;38(9):703–14.	130 (1,416)
Williams AM. Perceptual skill in soccer: Implications for talent identification and development. Journal of sport sciences [Internet]. 2000;18(9):737–50. Available from: http://www.tandf.co.uk/journals	87 (771)
Vaeyens R, Malina RM, Janssens M, Van Renterghem B, Bourgois J, Vrijens J, et al. A multidisciplinary selection model for youth soccer: The Ghent Youth Soccer Project. Br J Sports Med. 2006 Nov;40(11):928–34.	60 (754)
**Top 3 cited business article**	
Arthur W, Day EA, McNelly TL, Edens PS. A meta-analyses of the criterion-related validity of assessment center dimensions. Pers Psychol. 2003 Mar;56(1):125–53.	56 (645)
Spychalski AC, Quiñones MA, Gaugler BB, Pohley K. A survey of assessment center üractices in organizations in the united states. Pers Psychol [Internet]. 1997;50(1):71–90. Available from: https://onlinelibrary.wiley.com/doi/10.1111/j.1744-6570.1997.tb00901.x	44 (320)
Klimoski R, Brickner M. Why do assessment centers work? The puzzle of assessment center validity. Pers Psychol. 1987;40(2):243–60. Available from: https://onlinelibrary.wiley.com/doi/10.1111/j.1744-6570.1987.tb00603.x	41 (361)

Number in brackets represent the number of citations on GoogleScholar (May 2024) as a comparison of their relative proportion.

**Table 5:** Top 10 researchers in talent selection ranked by number of publications in scope.

**Rank**	**Author**	**Publications in articles**	**Fractionised publication**	**Main field**	**h-index**	**m-index**	**Total citations (highest number of databases SCOPUS, GS, and WOS)**	**First publication (earliest of both databases)**
1	Lievens, F.	29	12.37	Business	91	3.64	28,567	1998
2	Lenoir, M.^*^	21	3.79	Sport	51	1.82	9,277	1995
3	Fransen, J.	18	3.31	Sport	31	2.82	3,661	2012
4	Vaeyens, R.	18	3.22	Sport	51	2.31	12,690	2001
5	Kleinmann, M.	17	4.26	Business	42	1.27	5,101	1990
6	Pion, J.	17	2.89	Sport	29	0.91	3,406	1991
7	Baker, J.	15	4.57	Sport	81	2.7	24,864	1993
8	Melchers, K.G.	14	3.91	Business	31	1.41	3,374	2001
9	Robertson, S.	14	3.8	Sport	39	3.55	4,848	2012
10	Woods, C.T.	14	2.93	Sport	29	1.38	2,554	2002

Authors are ranked by the number of publications within the scope. Total publications (highest number), total citations (highest number) and first publication (earliest year) as found in GoogleScholar and SCOPUS database.

^*^no Google Scholar metrics were available and SCOPUS measures were used, which may underestimate characteristics. A full list of the top 40 authors can be found in the Supplementary material.

## 4 Discussion

The primary objective of this investigation was to systematically draw a representative picture of the extent of the scholarly linkage between talent selection literature in sports and business. The main findings of the CNA reveal a clear disconnection between the two contexts, with neither a direct nor an indirect path through the fundamental discipline of psychology. Although both contexts show similar fundamental goals (Ployhart et al., 2017; Williams and Reilly, 2000) sports and business form distinct clusters, with the business cluster further separated into two smaller clusters. The first of the two clusters seems to focus on psychometric testing and selection methods, while the second cluster seems to focus on talent management. For sports, only one cluster was found. They reveal that each field appears to develop its knowledge independently. Based on these findings, we conclude that there is limited awareness of potentially relevant literature from the other context. Notably, however, the majority of cross-contextual links have emerged only since 2018, highlighting a rising interest in cross-contextual integration and reflecting the increasing importance of bridging the two domains.

### 4.1 Similarities between selection in sports and business

Similarities between the two contexts can be found in psychological aspects that serve as predictors of performance, such as personality or motivation [e.g., Ivarsson et al. (2020) in sports; Baruch et al. (2004) in business]. In both contexts, these (Gagné, 2010) factors are illustrated in theoretical models, such as the Differentiated Model of Giftedness and Talent (Roberts et al., 2019), which includes intrapersonal factors like personality and motivation as catalysts in the developmental process from giftedness to talent. Within the work setting Cortina and Luchman (2013); based on Campbell et al. (1993) include personality factors as well as motivation as a mediator between abilities and dispositional traits on job performance. Moreover, empirical findings support the importance of psychological attributes in both contexts. In sports, Allen et al. (2013) identified personality traits as significant discriminators between elite and non-elite athletes (Klimoski and Brickner, 1987), and Spychalski et al. (1997) showed in their systematic review a positive relationship between the achievement component, hope for success and future performance. Parallel evidence exists in the business literature, where Barrick and Mount (1991) demonstrated significant relationships between the personality dimension conscientiousness and job performance. At the same time, Grant (2008) has shown that intrinsically motivated employees yield better job performance. Additionally, methodological foundations, such as the (Arthur and Villado, 2008; Sackett et al., 2022; Bergkamp et al., 2022b). Thus, even though significant similarities exist between the two contexts at both a construct level, such as recording psychological constructs (e.g., Eisenmann et al., 2020; Johnston and Baker, 2020) and a methodological level (e.g., Lievens et al., 2008; Collings and Mellahi, 2009), differences between sports and business remain, which may lead to the scholarly disconnection.

### 4.2 Disconnection between sports and business literature

The citation network analysis, as well as the co-citation network, reveal a clear disconnect between the two contexts on a literary level. This lack of a scholarly linkage may be due to several reasons: the leading reason may be the time point of selection. In sports, selection typically starts at the age of 12 years (Ford et al., 2020), whereas selection procedures in companies are implemented at substantially later developmental stages, predominantly post-adolescence (~18 years) and throughout distinct career phases (Dries, 2013). Although the experience of both groups, athletes and employees, may be comparable at the selection timing, suggesting potential as a relevant criterion, the predictive accuracy in athletic contexts is significantly constrained by maturational variance (Eisenmann et al., 2020). In contrast, selection in companies benefits from relative maturational stability, enabling more reliable assessment of current capabilities and short-term potential. This age-related factor may also contribute to differences in assessment focus: sports research frequently emphasizes developmental (Johnston et al., 2018; Vaeyens et al., 2008) and physical aspects (Baker et al., 2020) aspects, whereas business research predominantly concentrates on cognitive factors (Berry et al., 2024) and competency-based evaluations (Lievens et al., 2021).

Independent of these contextual demands, both show challenges in the prediction of performance (Bergkamp et al., 2019; Schmidt and Hunter, 1998). Empirical investigations in sport predominantly focus on individual relationships between performance and possible predictors (Johnston and Baker, 2020). Meanwhile, business research exhibits heterogeneous approaches to evaluating the predictive and incremental validity of theoretical constructs and methodological protocols (Berry et al., 2024; Schmidt and Hunter, 1998), which may further contribute to the lack of a well-established linkage between the two domains. The disconnection may also be justified by the fact that selection research in the sports context began decades after it started in business, resulting in a difference in the amount of research. Additionally, the evolution of selection methods has led to convergence between the contexts in recent years, albeit slowly. Nevertheless, in both contexts, a combination of objective and subjective measurements is now the dominant approach (Bar-Eli et al., 2024; Highhouse, 2008), with research aiming to find valid predictive constructs and methods for future performance (Schmidt and Hunter, 1998; Koz et al., 2012).

The analysis of citation patterns and the most prominent articles within the scope further reveal distinct emphases within the sports and business domains regarding talent identification methodologies. In the sports literature, highly cited (Vaeyens et al., 2008; Lefebvre et al., 2020; Williams, 2000; Vaeyens et al., 2006). Notable contributions include Vaeyens et al.'s (2008) theoretical framework, which synthesizes genetic and environmental factors in adolescent talent identification, emphasizing the critical integration of maturational status within dynamic talent identification and development protocols. Williams' (2000) examination of perceptual skill differentiation in soccer provides empirical evidence for skill-based talent identification metrics. In contrast, Vaeyens et al.s (2006) subsequent analysis of discriminative characteristics in youth soccer establishes the temporal dependency of talent indicators and advocates for adaptive identification methodologies. Collectively, these articles emphasize the multidimensional approach, including biological determinants as well as environmental influences. This approach holds implications for talent selection to be dynamic, age-specific and continuously adapted throughout developmental stages.

In the business domain, AC research dominates the most-cited literature, encompassing methodologically diverse approaches: meta-analytical validation studies (Arthur et al., 2003), empirical investigations of organizational implementation (Spychalski et al., 1997), and theoretical reviews (Collings and Mellahi, 2009). The highly cited articles of Spychalski et al. (1997) as well as Klimoski and Brickner (1987) reflect the importance of evidence-based selection practices in the business context and their practical implementation. The focus on AC reflects the approach of multi-method assessment while simultaneously addressing the research practice gap. The prevalent research from both contexts reveals a shared focus on multidimensional assessment. However, the sports context focuses mainly on developmental potential, while business prioritizes methodological validation and implementation strategies.

### 4.3 Connection between sports and business literature

Despite the above-mentioned apparent disconnection between both contexts characterized by differences in timing, developmental focus and methodological aspects, the CNA also reveals some connections. In addition to most of the literature, which appears to be unconnected, the analysis reveals six bidirectional references between sports and business literature, showing distinct patterns of methodological integration.

Within the sports context, the integration of business literature manifests through different dimensions: Johnston et al. (2018) are focusing on the understanding of talent identification and related issues, while Bergkamp et al. (2019) systematically incorporates selection-psychological principles into sport-specific talent identification. Den Hartigh et al. (2018) adapt the work of Lievens and Patterson (2011) to highlight the methodological differentiation between signs and samples approaches, especially regarding enhanced predictive validity of behavioral sampling in homogeneous elite populations, referring to the work context.

Conversely, within the business context, Terpstra et al. (2000) reference to Blakley et al. (1994) only includes the naming of physical ability tests and their relationship with job performance in a general introduction of selection methods. Nijs et al.'s (2014) integration of Bailey and Morley's (2006) framework demonstrates theoretical adaptation, particularly regarding deliberate conceptualization and expertise development paradigms. The paper integrates Bailey and Morley's findings with other literature on motivation and interests, suggesting that these factors, along with deliberate practice, are crucial for talent development. This holistic view underscores the importance of not only innate abilities but also the environmental and psychological factors that contribute to talent realization. Although some cross-contextual linkage is evident, the connection ranges from superficial adaptations and naming to more substantial theoretical adaptations. The latter holds potential for future integrated research. Future research in this direction may incorporate more profound knowledge and transfer methodological aspects.

Building upon these findings, they offer potential for future scholarly exploration, including the integration of methodological frameworks, particularly regarding psychological predictors, as well as the cross-contextual discussions about selection validity and the use of diverse methods. These aspects suggest an unexplored potential for theoretical synthesis and methodological transfer between the two contexts, which have thus far developed separately from each other within the common fundamental discipline of psychology.

The results only partially support the findings of a previous bibliometric analysis (Parra-Martinez and Wai, 2023), which shows an indirect connection through the fundamental discipline of psychology. Our co-citation network analysis reveals a disconnection, highlighting two distinct clusters and a limited number of co-citations between the two domains. This is surprising, as psychological research led to the disciplines of sport psychology as well as industrial-organizational psychology (Lievens et al., 2021; Gonzalez-Mulé et al., 2014).

## 5 Limitations

By including articles from the SCOPUS and Web of Science databases, we aimed to showcase the scientific discussion in both contexts as much as possible. Compared to Parra-Martinez and Wai (2023) the present study included a systematic literature search, followed by clearly defined inclusion and exclusion criteria and independent screening. Although this procedure resulted in a smaller number of included articles, it focused explicitly on the selection process in comparison to similar and related processes, such as talent identification and therefore expands the results of previous network analyses in this field. Even with a thorough screening process and the inclusion of a still high number of articles, it is likely that some articles may be missing due to their publication in other databases. On the other hand, the inclusion of articles that only briefly reference a topic might distort the analysis by inflating the perceived size of the clusters or creating false impressions of (inter-) connectedness. Additionally, the exclusion of several publication types, such as theses or book chapters, as well as limiting the literature search to specific subject areas, may exclude literature that could connect the two contexts. Furthermore, due to technical limitations in merging SCOPUS and Web of Science data within *bibliometrix*, only 893 of the initially identified 940 articles could be re-included in the co-citation network analysis. While this 5% reduction had no impact on the main CNA and therefore did not affect cross-contextual citations, it may have resulted in slightly smaller or less differentiated co-citation clusters. However, with >20,000 articles found in the initial literature search and including 940, respectively 893 articles in the final analysis, we believe that our findings are reliable and adequately represent the field.

Analytical limitations, such as text seniority (older articles seem to be more important), preferential attachment (citing established text to legitimize own work), self-citations, citation characteristics (no measurement of contextually relevant information) and inter-disciplinary norms (cross-contextual difference in citation patterns) should be considered when interpreting the results (Lefebvre et al., 2020). Given the extensive body of literature in this field, including related topics, the literature search was not exhaustive. Furthermore, by limiting the inclusion criteria to literature from psychology as a potential connecting discipline, other relevant fields such as genetics and statistics were not examined. The influence of these disciplines warrants further exploration, with an emphasis on identifying potential advantages and integrative opportunities for both domains.

## 6 Implications and future directions

The study aimed to assess the scholarly linkage between talent selection in sports and business in a representative manner. Based on the results, there remains cross-contextual potential. Regarding assessment methods, AC, as well as SJT, represent a substantial amount of research in the field of business research. Future research could consider testing these in sports talent selection. Both methods benefit from being highly position-specific and therefore have the potential to be tailored specifically to the demands of the sport or company. Furthermore, the utilization of psychological constructs (Roberts et al., 2019) within both contexts and the potential benefit for the other context could be assessed in future studies. Comparative method studies, i.e., transferring methods to other contexts, offer opportunities for future research and the sharing of knowledge. Lastly, focusing on development, both in sports and business (i.e., high-potential individuals, future leaders), may lead to the building of general theoretical frameworks.

In conclusion, our research reveals a complex integration of talent selection research across the sports and business context. Despite demonstrating fundamental psychological foundations and talent selection objectives for both, the CNA uncovers a scholarly disconnection. It has been shown that talent selection research is a prominent topic in sports and business literature, and therefore, a substantial amount of knowledge exists. Researchers in both contexts are encouraged to examine literature from other contexts for empirical, methodological and practical integration, resulting in a more advanced understanding. Especially the integration of methodological frameworks, particularly regarding psychological predictors, as well as the cross-contextual discussions about selection validity and the use of diverse methods, offers potential for further research. This may further integrate knowledge rather than prompting parallel development within the discipline of psychology.

## Data Availability

The datasets presented in this study can be found in online repositories. The names of the repository/repositories and accession number(s) can be found at: OSF https://osf.io/av45p/?view_only=4f8ff7ff00b24a2a8e5ba01b6a2ce152.
