# Supplementary material for: The (dis-)connection between selection research in sports and business literature – a citation network analysis
Source: Front Psychol. 2025 Jun 23;16:1604108. doi: 10.3389/fpsyg.2025.1604108 (PMC12236371; doi:10.3389/fpsyg.2025.1604108)
Supplement: Supplementary file 1 [file Table_1.pdf]

The (dis-)connection between sports and business literature – a citation network analysis

Supplementary material

Table S1. Top 10 Articles referenced within and between Sports and Business Literature

**Top 10 cited sports article**	**# citations**
Vaeyens R, Lenoir M, Williams AM, Philippaerts RM. Talent identification and development programmes in sport. Sports Medicine. 2008;38(9):703–14.	130(1,264)
Williams AM. Perceptual skill in soccer: Implications for talent identification and development. Journal of sport sciences [Internet]. 2000;18(9):737–50. Available from: http://www.tandf.co.uk/journals	87(726)
Vaeyens R, Malina RM, Janssens M, van Renterghem B, Bourgois J, Vrijens J, et al. A multidisciplinary selection model for youth soccer: The Ghent Youth Soccer Project. British Journal of Sports Medicine. 2006 Nov;40(11):928–34.	60(693)
Pearson DT, Naughton GA, Torode M. Predictability of physiological testing and the role of maturation in talent identification for adolescent team sports. Journal of Science and Medicine in Sport. 2006 Aug;9(4):277–87.	45(468)
Abbott A, Collins D. Eliminating the dichotomy between theory and practice in talent identification and development: Considering the role of psychology. Journal of Sports Sciences. 2004;22(5):395–408.	38(879)
Meylan C, Cronin J, Oliver J, Hughes M. Talent Identification in Soccer: The Role of Maturity Status on Physical, Physiological and Technical Characteristics. International Journal of Sports Science & Coaching. 2010;5(4):571–92.	35 (421)
Johnston K, Wattie N, Schorer J, Baker J. Talent identification in sport: A systematic review. Sports Med. 2018;48(1):97-109. doi:10.1007/s40279-017-0803-2	28(364)
Hoare GD, Warr CR. Talent identification and women's soccer: An Australian experience. Journal of Sports Sciences. 2000 Jan;18(9):751–8.	25(324)
Sherar LB, Baxter-Jones ADG, Faulkner RA, Russell KW. Do physical maturity and birth date predict talent in male youth ice hockey players? Journal of Sports Sciences. 2007;25(8):879-886. doi:10.1080/02640410600908001	25(432)
Vandendriessche JB, Vaeyens R, Vandorpe B, Lenoir M, Lefevre J, Philippaerts RM. Biological maturation, morphology, fitness, and motor coordination as part of a selection strategy in the search for international youth soccer players (age 15–16 years). Journal of Sports Sciences. 2012;30(15):1695-1703. doi:10.1080/02640414.2011.652654	24(267)
**Top 10 cited business article**	**# citations**
Arthur W, Day EA, Mcnelly TL, Edens PS. A meta-analysis of the criterion-related validity of assesment center dimensions. Personnel Psychology. 2003;56(1):125-153. doi:10.1111/j.1744-6570.2003.tb00146.x	56(624)

Spychalski AC, Quiñones MA, Gaugler BB, Pohley K. A survey of assessment center üractices in organizations in the united states. Personnel Psychology. 1997;50(1):71-90. doi:10.1111/j.1744-6570.1997.tb00901.x	44(309)
Klimoski R, Brickner M. Why do assessment centers work? The puzzle of assessment center validity. Personnel Psychology. 1987;40(2):243-260. doi:10.1111/j.1744-6570.1987.tb00603.x	41(338)
Lance CE, Foster MR, Gentry WA, Thoresen JD. Assessor cognitive processes in an operational assessment center. Journal of Applied Psychology. 2004;89(1):22-35. doi:10.1037/0021-9010.89.1.22	38(106)
Cable DM, Judge TA. Interviewers' perceptions of person–organization fit and organizational selection decisions. Journal of Applied Psychology. 1997;82(4):546-561. doi:10.1037/0021-9010.82.4.546	37(1284)
Woehr DJ, Arthur W. The Construct-Related Validity of Assessment Center Ratings: A Review and Meta-Analysis of the Role of Methodological Factors. Journal of Management. 2003;29(2):231-258. doi:10.1177/014920630302900206	31(241)
Lievens F, Chasteen CS, Day EA, Christiansen ND. Large-scale investigation of the role of trait activation theory for understanding assessment center convergent and discriminant validity. Journal of Applied Psychology. 2006;91(2):247-258. doi:10.1037/0021-9010.91.2.247	29(268)
Huffcutt AI, Conway JM, Roth PL, Stone NJ. Identification and meta-analytic assessment of psychological constructs measured in employment interviews. Journal of Applied Psychology. 2001;86(5):897-913. doi:10.1037/0021-9010.86.5.897	29 (646)
Kristof-Brown AL. Perceived applicant fit: Distinguishing between recruiters' perceptions of person–job and person–organization fit. Personnel Psychology. 2000;53(3):643-671. doi:10.1111/j.1744-6570.2000.tb00217.x	28(993)
Lievens F. Factors which improve the construct validity of assessment centers: A review. Int J Selection Assessment. 1998;6(3):141-152. doi:10.1111/1468-2389.00085	27(197)
**Business paper cited in sport literature**	**# citations**
Nijs S, Gallardo-Gallardo E, Dries N, Sels L. A multidisciplinary review into the definition, operationalization, and measurement of talent. Journal of World Business. 2014;49(2):180-191. doi:10.1016/j.jwb.2013.11.002	1
Robertson IT, Smith M. Personnel selection. J Occupat & Organ Psyc. 2001;74(4):441-472. doi:10.1348/096317901167479	1
Lievens F, Patterson F. The validity and incremental validity of knowledge tests, low-fidelity simulations, and high-fidelity simulations for predicting job performance in advanced-level high-stakes selection. Journal of Applied Psychology. 2011;96(5):927-940. doi:10.1037/a0023496	1
**Sport paper cited in business literature**	**# citations**
Blakley BR, Quiñones MA, Crawford MS, Jago IA. The validity of isometric strength tests. Personnel Psychology. 1994;47(2):247-274. doi:10.1111/j.1744-6570.1994.tb01724.x	1

Bailey R, Morley D. Towards a model of talent development in physical education. Sport, Education and Society. 2006;11(3):211-230. doi:10.1080/13573320600813366	1
Vaeyens R, Güllich A, Warr CR, Philippaerts R. Talent identification and promotion programmes of olympic athletes. Journal of Sports Sciences. 2009;27(13):1367-1380. doi:10.1080/02640410903110974	1

Note: [1] number of citations shows the number within the network within the corresponding field; numbers in brackets show the number of citations of google scholar as a reference number (04.2023).
[2] all papers between categories were only cited ones between networks

Table S2. Centrality measures for top 5 articles

Ranking	Score	Category	
			**Degree-Centrality**
1	143	sport	Vaeyens R, Lenoir M, Williams AM, Philippaerts RM. Talent identification and development programmes in sport. Sports Medicine. 2008;38(9):703–14.
2	88	sport	Williams AM. Perceptual skill in soccer: Implications for talent identification and development. Journal of sport sciences [Internet]. 2000;18(9):737–50. Available from: http://www.tandf.co.uk/journals
3	70	business	Arthur W, Day EA, Mcnelly TL, Edens PS. A meta-analysis of the criterion-related validity of assesment center dimensions. Personnel Psychology. 2003;56(1):125-153. doi:10.1111/j.1744-6570.2003.tb00146.x
4	65	sport	Vaeyens R, Malina RM, Janssens M, van Renterghem B, Bourgois J, Vrijens J, et al. A multidisciplinary selection model for youth soccer: The Ghent Youth Soccer Project. British Journal of Sports Medicine. 2006 Nov;40(11):928–34.
5	52	sport	Pearson DT, Naughton GA, Torode M. Predictability of physiological testing and the role of maturation in talent identification for adolescent team sports. Journal of Science and Medicine in Sport. 2006 Aug;9(4):277–87.
			**Indegree-Centrality**
1	130	sport	Vaeyens R, Lenoir M, Williams AM, Philippaerts RM. Talent identification and development programmes in sport. Sports Medicine. 2008;38(9):703–14.
2	87	sport	Williams AM. Perceptual skill in soccer: Implications for talent identification and development. Journal of sport sciences [Internet]. 2000;18(9):737–50. Available from: http://www.tandf.co.uk/journals
3	60	sport	Vaeyens R, Malina RM, Janssens M, van Renterghem B, Bourgois J, Vrijens J, et al. A multidisciplinary selection model for youth soccer: The Ghent Youth Soccer Project. British Journal of Sports Medicine. 2006 Nov;40(11):928–34.
4	56	business	Arthur W, Day EA, Mcnelly TL, Edens PS. A meta-analysis of the criterion-related validity of assesment center dimensions. Personnel Psychology. 2003;56(1):125-153. doi:10.1111/j.1744-6570.2003.tb00146.x
5	45	sport	Pearson DT, Naughton GA, Torode M. Predictability of physiological testing and the role of maturation in talent identification for adolescent team sports. Journal of Science and Medicine in Sport. 2006 Aug;9(4):277–87.
			**Eigenvector-Centrality**
1		sport	Vaeyens R, Lenoir M, Williams AM, Philippaerts RM. Talent identification and development programmes in sport. Sports Medicine. 2008;38(9):703–14.
2		sport	Williams AM. Perceptual skill in soccer: Implications for talent identification and development. Journal of sport sciences [Internet]. 2000;18(9):737–50. Available from: http://www.tandf.co.uk/journals
3		sport	Vaeyens R, Malina RM, Janssens M, van Renterghem B, Bourgois J, Vrijens J, et al. A multidisciplinary selection model for youth soccer: The Ghent Youth Soccer Project. British Journal of Sports Medicine. 2006 Nov;40(11):928–34.

4	sport	Abbott A, Collins D. Eliminating the dichotomy between theory and practice in talent identification and development: Considering the role of psychology. Journal of Sports Sciences. 2004;22(5):395–408.
5	sport	Pearson DT, Naughton GA, Torode M. Predictability of physiological testing and the role of maturation in talent identification for adolescent team sports. Journal of Science and Medicine in Sport. 2006 Aug;9(4):277–87.
	**Betweenness-Centrality**	
1	business	Robertson IT, Smith M. Personnel selection. J Occupat & Organ Psyc. 2001;74(4):441-472. doi:10.1348/096317901167479
2	sport	Bergkamp TLG, Niessen ASM, den Hartigh RJR, Frencken WGP, Meijer RR. Methodological issues in soccer talent identification research. Sports Medicine. 2019 Sep 1;49(9):1317–35.
3	sport	Vaeyens R, Lenoir M, Williams AM, Philippaerts RM. Talent identification and development programmes in sport. Sports Medicine. 2008;38(9):703–14.
4	business	Arthur W, Day EA, Mcnelly TL, Edens PS. A meta-analysis of the criterion-related validity of assesment center dimensions. Personnel Psychology. 2003;56(1):125-153. doi:10.1111/j.1744-6570.2003.tb00146.x
5	sport	den Hartigh RJR, Niessen ASM, Frencken WGP, Meijer RR. Selection procedures in sports: Improving predictions of athletes' future performance. Vol. 18, European Journal of Sport Science. Taylor and Francis Ltd.; 2018. p. 1191–8.

Table S3. Top 40 authors

Rank	Author	Pub-lications in articles	fractionized publication	main field	*h-index*	*m-index*	*total citations**	*first publication (earlierst of both databases)*
1	Lievens, F.	29	12,37	business	91	3,64	28,567	1998
2	Lenoir, M. *	21	3,79	sport	51	1,82	9,277	1995
3	Fransen, J.	18	3,31	sport	31	2,82	3,661	2012
4	Vaeyens, R.	18	3,22	sport	51	2,31	12,690	2001
5	Kleinmann, M.	17	4,26	business	42	1,27	5,101	1990
6	Pion, J.	17	2,89	sport	29	0,91	3,406	1991
7	Baker, J.	15	4,57	sport	81	2,7	24,864	1993
8	Melchers, K.G.	14	3,91	business	31	1,41	3,374	2001
9	Robertson, S.	14	3,8	sport	39	3,55	4,848	2012
10	Woods, C.T.	14	2,93	sport	29	1,38	2,554	2002
11	Philippaerts, R.M. *	14	2,4	sport	54	1,93	9,265	1995
12	Schorer, J.	12	2,85	sport	43	1,48	6,425	1994
13	Elferink-Gemser, M.T.	12	2,81	sport	50	2,63	8,870	2004
14	Conzelmann, A.	10	3,67	sport	35	1,03	3,987	1989
15	König, C.J.	10	2,95	business	46	2,19	9,145	2002
16	Till, K.	10	2,55	sport	49	3,27	8,496	2008
17	Faber, I.	10	2,09	sport	17	1	1,101	2006
18	Zuber, C. *	9	3,33	sport	12	1,5	310	2015
19	Thornton, G.C. *	9	2,92	business	24	0,43	2,234	1967
20	Hoffmann, B.J.	9	2,91	business	37	1,61	13,435	2000
21	Deprez, D. *	9	1,39	sport	19	1,72	1,208	2012
22	Sackett, P.R.	8	4,17	business	84	1,71	33,587	1974
23	Williams, A.M.	8	3,09	sport	113	3,23	54,691	1988
24	Cobley, S.	8	1,69	sport	51	2,32	10,130	2001
25	Ones, D.S.	7	2,92	business	79	2,47	34,355	1991
26	Collins, D. *	7	2,75	sport	49	1,48	8,434	1990
27	Ingold, P.V.	7	1,89	business	12	1,5	552	2015
28	Pyne, D.B.	7	1,87	sport	95	2,02	30,159	1976
29	Visscher, C. *	7	1,63	sport	48	2,09	6883	2000
30	Roth, P.L.	7	1,59	business	56	1,47	20,643	1985
31	Raynor, A.J.	7	1,57	sport	17	0,5	1,323	1989
32	Bennett, K.J.	7	1,44	sport	16	1,77	2,005	2014
33	Malina, R.M.	7	1,3	sport	124	1,97	73,878	1960
34	Furnham, A.	6	2,37	business	183	4,16	155,466	1979
35	Born, M.	6	2,32	business	47	1,31	8,373	1987
36	Höner, O.	6	2,28	sport	27	1,08	2,430	1998
37	Anderson, N	6	2,15	business	77	2,08	35,884	1986
38	Arthur, W.	6	1,92	business	52	1,37	15,343	1985
39	Goffin, R.D.	6	1,87	business	37	1,09	8,733	1988
40	Jackson, D.J. *	6	1,73	business	14	0,66	419	2002

Note: (highest number of databases SCOPUS, GS, and WOS)*

Table S4. Articles not included in Co-Citation Network Analysis (*n* = 47)

**Article**	**category**
Arvidsson, J., & Haglund, E. (2019). Jump height as performance indicator for the selection of youth football players to national teams. *The Journal of Sports Medicine and Physical Fitness, 59*(10), 1669-1675.	sport
Boccia, G., Cardinale, M., & Brustio, P. R. (2020). World-class sprinters' careers: Early success does not guarantee success at adult age. *International Journal of Sports Physiology and Performance, 16*(3), 367-374.	sport
Bullock, N., Gulbin, J. P., Martin, D. T., Ross, A., Holland, T., & Marino, F. (2009). *Talent identification and deliberate programming in skeleton: Ice novice to Winter Olympian in 14 months. Journal of sports sciences, 27*(4), 397-404.	sport
Camacho-Cardenosa, A., Camacho-Cardenosa, M., González-Custodio, A., Martínez-Guardado, I., Timón, R., Olcina, G., & Brazo-Sayavera, J. (2018). Anthropometric and physical performance of youth handball players: *The role of the relative age. Sports, 6*(2), 47.	sport
Chytiri, A. P., Filippaios, F., & Chytiris, L. (2018). Hotel recruitment and selection practices: The case of the Greek Hotel Industry. *International Journal of Organizational Leadership, 7*, 324-339.	business
Dondolo, V., & Chinyamurindi, W. T. (2018). Impression management within the recruitment interview: Narratives of employees at a South African higher education institution. *SA Journal of Industrial Psychology, 44*(1), 1-7.	business
Goto, H., Morris, J. G., & Nevill, M. E. (2019). Influence of biological maturity on the match performance of 8-to 16-year-old, elite, male, youth soccer players. *The Journal of Strength & Conditioning Research, 33*(11), 3078-3084.	sport
Gryko, K., Kopiczko, A., Mikołajec, K., Stasny, P., & Musalek, M. (2018). Anthropometric variables and somatotype of young and professional male basketball players. *Sports, 6*(1), 9.	sport
Haycraft, J. A., Kovalchik, S., Pyne, D. B., & Robertson, S. (2017). Physical characteristics of players within the Australian Football League participation pathways: a systematic review. *Sports medicine-open, 3*(1), 1-16.	sport
Hohmann, A., Siener, M., & He, R. (2018). Prognostic validity of talent orientation in soccer. *German Journal of Exercise and Sport Research, 48*(4), 478-488.	sport

Jacob, Y., Spiteri, T., Hart, N. H., & Anderton, R. S. (2018). The potential role of genetic markers in talent identification and athlete assessment in elite sport. *Sports, 6*(3), 88.	sport
Kalinski, S. D., Jelaska, I., & Knežević, N. (2017). Age effects among elite male gymnasts. *Acta Kine, 11*(2), 84-89.	sport
Koch, T., Gerber, C., & De Klerk, J. J. (2018). The impact of social media on recruitment: Are you LinkedIn?. *SA Journal of Human Resource Management, 16*(1), 1-14.	business
Köklü, Y., Arslan, Y., & Alemdaroğlu, U. (2017). Evidence of the relative age effect in youth soccer players from Turkey. *Kinesiologia Slovenica, 23*(2), 33-43.	sport
Lance, C. E., Lambert, T. A., Gewin, A. G., Lievens, F., & Conway, J. M. (2004). Revised estimates of dimension and exercise variance components in assessment center postexercise dimension ratings. *Journal of Applied Psychology, 89*(2), 377.	business
Landers, R. N., & Marin, S. (2021). Theory and technology in organizational psychology: A review of technology integration paradigms and their effects on the validity of theory. *Annual Review of Organizational Psychology and Organizational Behavior, 8*, 235-258.	business
Langer, M., König, C. J., & Fitili, A. (2018). Information as a double-edged sword: The role of computer experience and information on applicant reactions towards novel technologies for personnel selection. *Computers in Human Behavior, 81*, 19-30.	business
Langer, M., König, C. J., & Hemsing, V. (2020). Is anybody listening? The impact of automatically evaluated job interviews on impression management and applicant reactions. *Journal of Managerial Psychology*.	business
Mahfoozi, A., Salajegheh, S., Ghorbani, M., & Sheikhi, A. (2018). Developing a talent management model using government evidence from a large-sized city, Iran. *Cogent Business & Management, 5*(1), 1449290.	business
Marcus, B., Goldenberg, J., Fine, S., Hummert, H., & Traum, A. (2020). Self-presentation in selection settings: The case of personality tests. *Journal of Business and Psychology, 35*(5), 557-571.	business
Markovic, G., & Mikulic, P. (2011). Discriminative ability of the yo-yo intermittent recovery test (level 1) in prospective young soccer players. *The Journal of Strength & Conditioning Research, 25*(10), 2931-2934.	sport
Medina, A., & Francis, A. J. (2015). What are the characteristics that software development project team members associate with a good project manager?. *Project Management Journal, 46*(5), 81-93.	business

Memon, M., Ahmed, F., Qureshi, M. A., & Brohi, N. A. (2018). Effectiveness of psychometric testing in recruitment process. *Journal of Organizational Behavior Research, 3*(1), 293-306.	business
Mwila, N. K., & Turay, M. I. S. (2018). Augmenting talent management for sustainable development in Africa. *World Journal of Entrepreneurship, Management and Sustainable Development, 14*(1), 41-49.	business
Norjali, R., Mostaert, M., Pion, J., & Lenoir, M. (2018). Anthropometry, physical performance, and motor coordination of medallist and non-medallist young fencers. *Archives of Budo, 14*, 33-40.	sport
Nortje, L., Dicks, M., Coopoo, Y., & Savelsbergh, G. J. (2014). Put your money where your mouth is: verbal self-reported tactical skills versus on-line tactical performance in soccer. *International Journal of Sports Science & Coaching, 9*(2), 321-334.	sport
Pao, H. W., Lee, C. Y., Chung, P. H., & Wu, H. L. (2018). Hiring decisions on certified manpower: The resource dependence and social contagion views of institutional innovators. *Journal of Advances in Management Research*.	business
Poppleton, W. L., & Salmoni, A. W. (1991). Talent identification in swimming. *Journal of Human Movement Studies, 20*(2), 85-100.	sport
Prieto-Ayuso, A., Pastor-Vicedo, J. C., & Contreras-Jordán, O. (2017). Content validity and psychometric properties of the nomination scale for identifying football talent (NSIFT): *Application to coaches, parents and players. Sports, 5*(1), 2.	sport
Puchert, J. I., Dodd, N., & Viljoen, K. L. (2017). Secondary education as a predictor of aptitude: Implications for selection in the automotive sector. *SA Journal of Industrial Psychology, 43*(1), 1-13.	business
Rajaprasad, S. V. S. (2018). Selection of safety officers in an indian construction organization by using grey relational analysis. *Independent Journal of Management & Production, 9*(1), 97-110.	business
Ré*, A. H., Cattuzzo, T. M., Santos, F. M., & Monteiro, C. B. (2014). Anthropometric characteristics, field test scores and match-related technical performance in youth indoor soccer players with different playing status. *International Journal of Performance Analysis in Sport, 14*(2), 482-492.	sport
Schmid, M. J., Conzelmann, A., & Zuber, C. (2021). Patterns of achievement-motivated behavior and performance as predictors for future success in rowing: A person-oriented study. *International journal of sports science & coaching, 16*(1), 101-109.	business
Silva, M. C., Figueiredo, A. J., Simoes, F., Seabra, A., Natal, A., Vaeyens, R., ... & Malina, R. M. (2010). Discrimination of u-14 soccer players by level and position. *International journal of sports medicine, 31*(11), 790-796.	sport

Slavić, A., Bjekić, R., & Berber, N. (2017). The role of the internet and social networks in recruitment and selection process. *Strategic Management-International Journal of Strategic Management and Decision Support Systems in Strategic Management, 23*(3).	business
Smither, J. W., Reilly, R. R., Millsap, R. E., AT&T, K. P., & Stoffey, R. W. (1993). Applicant reactions to selection procedures. *Personnel psychology, 46*(1), 49-76.	business
Till, K., Cobley, S., O'Hara, J., Brightmore, A., Cooke, C., & Chapman, C. (2011). Using anthropometric and performance characteristics to predict selection in junior UK Rugby League players. *Journal of Science and Medicine in Sport, 14*(3), 264-269.	sport
Till, K., Cobley, S., O'Hara, J., Chapman, C., & Cooke, C. (2013). A longitudinal evaluation of anthropometric and fitness characteristics in junior rugby league players considering playing position and selection level. *Journal of Science and Medicine in Sport, 16*(5), 438-443.	sport
Till, K., Morley, D., O'Hara, J., Jones, B. L., Chapman, C., Beggs, C. B., ... & Cobley, S. (2017). A retrospective longitudinal analysis of anthropometric and physical qualities that associate with adult career attainment in junior rugby league players. *Journal of science and medicine in sport, 20*(11), 1029-1033.	sport
Till, K., Scantlebury, S., & Jones, B. (2017). Anthropometric and physical qualities of elite male youth rugby league players. *Sports Medicine, 47*(11), 2171-2186.	sport
Schorer, J., Rienhoff, R., Fischer, L., & Baker, J. (2017). Long-term prognostic validity of talent selections: comparing national and regional coaches, laypersons and novices. *Frontiers in psychology, 8*, 1146.	sport
Schorer, J., Faber, I., Koopmann, T., Büsch, D., & Baker, J. (2020). Predictive value of coaches' early technical and tactical notational analyses on long-term success of female handball players. *Journal of Sports Sciences, 38*(19), 2208-2214.	sport
Scullen, S. E., & Meyer, B. C. (2014). More applicants or more applications per applicant? A big question when pools are small. *Journal of Management, 40*(6), 1675-1699.	business
Tlaiss, H. (2020). Exploring talent management in practice: an Arab country-specific empirical investigation. Employee Relations: *The International Journal*.	business
Tziner, A., Ronen, S., & Hacohen, D. (1993). A four-year validation study of an assessment center in a financial corporation. *Journal of Organizational Behavior, 14*(3), 225-237.	business
Waheed, A., & Yang, J. (2019). Effect of Prejudice and References on Employee Selection Process: Empirical Evidence from Pakistan. *Global Business Review, 20*(6), 1344-1360.	business

Wilson, R. S., Smith, N. M., Bedo, B. L. S., Aquino, R., Moura, F. A., & Santiago, P. R. P. (2020). Technical skill not athleticism predicts an individual’s ability to maintain possession in small-sided soccer games. *Science and Medicine in Football, 4*(4), 305-313.	sport
